# Vital Sign Monitoring in Car Seats Based on Electrocardiography, Ballistocardiography and Seismocardiography: A Review

**DOI:** 10.3390/s20195699

**Published:** 2020-10-06

**Authors:** Michaela Sidikova, Radek Martinek, Aleksandra Kawala-Sterniuk, Martina Ladrova, Rene Jaros, Lukas Danys, Petr Simonik

**Affiliations:** 1 Faculty of Electrical Engineering and Computer Science, VSB-Technical University of Ostrava, 17 Listopadu 15, 70800 Ostrava, Czech Republic; martina.ladrova@vsb.cz (M.L.); rene.jaros@vsb.cz (R.J.); lukas.danys@vsb.cz (L.D.); petr.simonik@vsb.cz (P.S.); 2 Faculty of Electrical Engineering, Automatic Control and Informatics, Opole University of Technology, Proszkowska 76, 45-758 Opole, Poland; a.kawala-sterniuk@po.edu.pl

**Keywords:** vital sign monitoring, sensors, car seats, electrocardiography, ballistocardiography, seismocardiography, electroencephalography, signal processing

## Abstract

This paper focuses on a thorough summary of vital function measuring methods in vehicles. The focus of this paper is to summarize and compare already existing methods integrated into car seats with the implementation of inter alia capacitive electrocardiogram (cECG), mechanical motion analysis Ballistocardiography (BCG) and Seismocardiography (SCG). In addition, a comprehensive overview of other methods of vital sign monitoring, such as camera-based systems or steering wheel sensors, is also presented in this article. Furthermore, this work contains a very thorough background study on advanced signal processing methods and their potential application for the purpose of vital sign monitoring in cars, which is prone to various disturbances and artifacts occurrence that have to be eliminated.

## 1. Introduction

**Figure 1:**
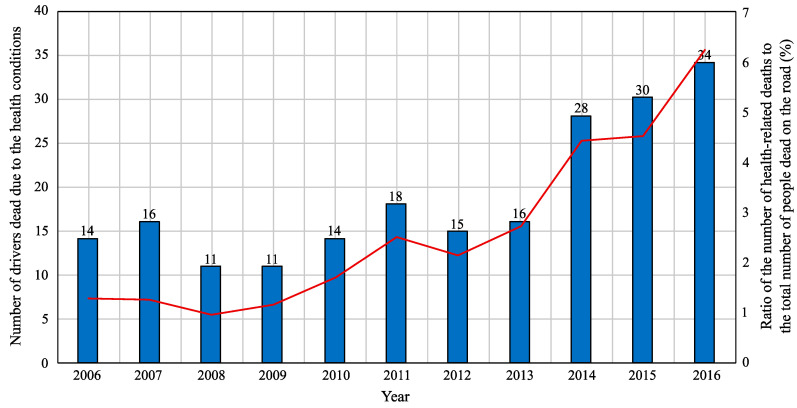
Number of drivers who died while driving due to health reasons, according to statistics from the Police of the Czech Republic.

**Figure 2:**
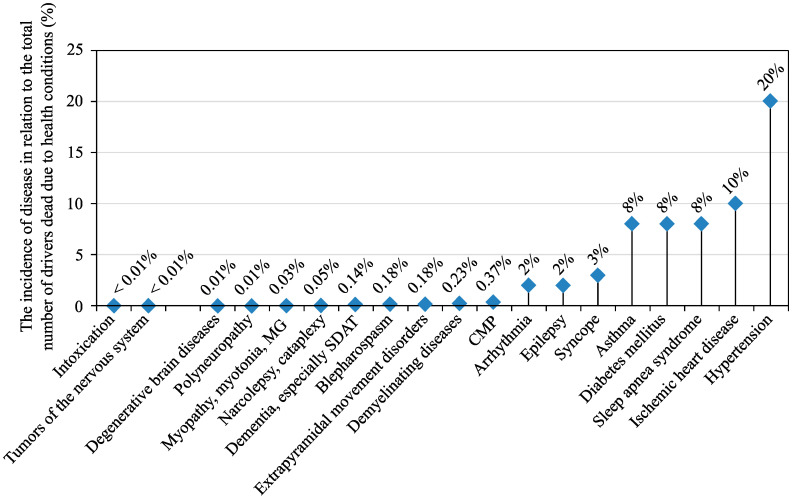
The incidence of diseases having a major impact.

In the future, the majority of vehicles will probably be fully autonomous, so the driver will play a less significant role than today. The transition to autonomous vehicles is an ongoing process, and the era of fully autonomous vehicles is slowly approaching. Until then, it will be necessary to continuously monitor and warn drivers, who have to stay alert, no matter the situation on the road [1,2,3,4,5]. Even a short diversion caused by a reflection in the rear-view mirror can affect the whole journey and lead to major accidents [5,6]. Long journeys also lead to fatigue; therefore, it can be very challenging to keep constant attention on the road [7,8]. As living standards increase, so does the number of vehicles and thus the density of traffic and the number of traffic accidents [9,10]. Numerous researchers are currently working on various driver response systems, which can measure various life functions of the driver in order to monitor his/her alertness [2,6,7,8,11,12,13,14,15,16]. These systems mainly detect fatigue, stress and the driver’s health condition. Figure 1 and Figure 2 represent the statistics from Czech roads. In Figure 1, you can see the increasing number of drivers who died due to health-related conditions in comparison to the total number of people that died on roads (according to the statistics of the Police of the Czech Republic). In addition, Figure 2 shows the most common diseases that have a major impact on mortality on Czech roads.

To design a proper countermeasure, it is necessary to monitor and recognize the approaching critical moment when the driver loses the attention or even total control of the vehicle. These critical situations can be predicted by various bodily responses such as faster eye movement and blinking, electrocardiogram changes, specific facial expressions and muscles movements, certain muscle activities, hand vibrations, fluctuations of blood pressure, increase in heart rate, changes in skin conductivity resistance, respiratory rate or brain activity, alcohol levels in blood, changes in sugar level and oxygen in blood or also by sudden changes in skin temperature. Existing systems cannot predict these states, as they are only capable of detecting the current state. A partially or fully automated system that can respond to the previously mentioned conditions offers a significantly faster response time and can save human lives, especially when connected to the various autonomous vehicular systems.

One way to divide sensors used to monitor the driver’s condition is to specify their deployed location. To ensure ongoing operation, the driver has to be connected and monitored either directly (through contact) or by cameras and other wireless sensors (contactless). Each approach has different advantages and disadvantages. Contact sensors tend to cause slight or major discomfort to the driver, as they require their direct placement on body/skin. Based on their construction, these sensors can also interfere with the driver’s actions. From the practical perspective, contact monitoring systems, such as cables or probes, are not appropriate, mainly due to the driver’s discomfort. However, in certain critical situations, they can offer more reliable data than contactless variants. On the other side stands various contactless technologies. These technologies do not interfere with the driver, can operate completely independently and are not limited by bodily proportions. Table 1 presents a summary of sensors with their division into the sensors that has to be attached to the driver’s body and others mounted as a part of the vehicle.

The implementation of car-mounted sensor is nowadays still a very challenging task due to the occurrence of numerous interfering artifacts, which are mainly caused by the driving itself. This artifact has to be eliminated or at least partially compensated.

This paper will mainly focus on detailed analysis of various sensors mounted into car seats, which despite significance of this topic, have not been discussed in depth. In addition, it also includes a detailed overview of the most recent and the most popular methods for vital sign monitoring in vehicles (e.g., camera systems, sensors in the steering wheel). Furthermore, a summary of various disturbances occurring during monitoring via a car seat and an overview of methods for disturbance elimination are presented and thoroughly described.

### Background Study on Vital Sign Monitoring Systems

The ongoing demographic change and slowly increasing life expectancy will lead to an increasing number of older car drivers [1,5,17,18]. That is a reason for the necessity of medical support in the automotive industry, which is expected to attract further attention from insurance companies, healthcare providers or emergency services. Advanced health monitoring systems in vehicles are, therefore, basically a necessary addition and are expected to attract further attention from insurance companies, healthcare providers or emergency services. These systems can basically react in two ways. The first one is a system which would estimate the driver’s capabilities and detect acute health problems. A set of complex medical sensors could detect a critical condition and initiate appropriate countermeasures, ranging from adjustments to driving (e.g., secure autopilot) to emergency services (e.g., a direct call to the hospital). The second application is based around sensory systems which monitor and regularly acquire the driver´s vital signs to initiate split-second automatized decisions. In addition, such system could be used in a broader context as part of a home health monitoring system, further expanding coverage and connectivity.

Four primary vital signs are nowadays used for bedside monitoring and assessment of patient´s status: heart rate (HR), respiratory rate (RR), blood pressure (BP) and body temperature (BP) [19,20,21,22,23,24]. These methods usually require close physical interaction with the examined patient (cables, electrodes, cuffs, etc.), which makes them suitable for clinical environments but limits their deployment to non-hospital applications [19,22]. In addition, in automotive applications, there are significant motion artifacts caused by sudden acceleration/deceleration as they cause sudden changes in the distance change between one (or both) electrode surface and the body. This problem still remains and is difficult to solve [1,25,26].

The development of technology for unobtrusive and non-contact vital sign monitoring has brought a large amount of attention in recent years [22,23,24,27]. Active driver status monitoring has been a major research topic for a long time, as it has a significant impact on road safety and accident statistics. Various physiological sensors based on electrocardiogram (ECG) and electroencephalogram (EEG) are nowadays frequently used in numerous studies [28,29].

For example, M. Walter et al. [11] used an alternative approach, placed the ECG electrodes on the human back and tried to systematically optimize the electrolytic effect of said electrodes on the human back. In addition, various electrode deployment positions and combinations were studied as well. The amplitude of the QRS complex was measured, since it is used as a sensitivity indicator. The measured results clearly confirmed that the angled electrode arrangement reached a significantly higher sensitivity in comparison to the horizontal positioning. In fact, this outcome is not very surprising since the typical electrical heart axis for the QRS complex can range from −30 ° to +90 ° (with respect to the horizontal axis when measured frontally on the chest). Typical values are between 30 ° and 60 °, corresponding to the obtained measurements.

For many years, scientists have focused on detection of stress, drowsiness, etc. [30,31,32]. These methods mainly employ video cameras and conventional imaging technologies, relying often on eye blinking frequency or the percentage of closed eyelids. [31,33,34].

The level of drowsiness can be also monitored by the heart rate variability (HRV) parameter, reflecting the everchanging balance between sympathetic and parasympathetic activity [35]. Slower HRV rhythms (LF) are a sign of increased sympathetic or lower vagal activity, thus pointing to the driver´s alertness, while faster HRV rhythms (HF) are a sign of lower sympathetic or increased parasympathetic and vagal activity indicating weariness and sleep [36]. It is important to mention the work of E. Michail et al. [37], which demonstrated that power spectral analysis of the driver’s heart rate can report and thus also predict driving errors caused by sleepiness. Both ECG and EEG data were obtained from subjects deprived of sleep who were exposed to off-road driving conditions. Lower ratio of low-frequency and high-frequency components (LF/HF) and lower LF values were reported when driving errors occurred.

Currently, there are multiple sensors located on various locations (e.g., in the steering wheel [1,38,39,40] or sensors which focus on the eyes in order to monitor drivers’ activity [3,4,17,41]. One such system which was integrated into a car seat was developed by researchers from the Nottingham Trent University in UK. It provides driver drowsiness detection with immediate sound alert response [12,13]. Sensors used to monitor vital signs in a car are used for both safety and efficiency purposes, albeit their location may differ depending on the specific project [1,11,13].

A fabric-based sensor system integrated inside the car seat could detect fluctuations in measured data (the driver’s focus and alertness) and advise the driver to immediately stop driving. If the driver ignores the signals, the system will activate cruise control or lane departure technology in order to prevent road accidents. The system can even send information to the remote-control center via a wireless network to take further actions.

For this study purpose, three measurement methods of vital signs contactless monitoring were investigated. In particular, the authors focused on capacitive ECG (cECG) monitoring, mechanical motion analysis (BCG—ballistocardiography) with the application of piezo-foils and inductive impedance monitoring. Further investigation was carried out for the purpose of their future integration into car seats. It is important to mention that all the three sensor techniques are not dependent on electrically conductive contact with the human body (driver) but require defined mechanical boundary conditions (stable distances, or in the case of BCG, a friction connection).

Unfortunately, deployment of car seats as a source for vital sign monitoring is not a widespread topic. Therefore, any additional research of this interesting topic may play a very important role for both professional and ordinary drivers, as the security on roads is becoming an increasingly relevant topic [1,2,3,4]. Therefore, modified car seats, capable of measuring and monitoring the vital signs of the driver, could be an integral part of every car and would definitely improve safety and reaction time during critical scenarios [4,41,42].

Because built-in wireless sensors are a very important part of computer networks, the technologies inside the car can help improve safety monitoring as well as health [42]. Leonhardt et al. [1] reported in detail in his study physical principles and specific boundary conditions related to the automotive vehicle integration.

A very widespread topic is the integration of wireless signal transmission modules into vehicles and peripherals. Modern wireless modules integrated in car seats can offer a great advantage over conventional cable connections, as many modern vehicle seats face problems due to limited cable routing space. When the previously discussed non-contact monitoring of cardiac function is used, the passenger’s cECG and BCG signal can be measured simultaneously. Due to various artifacts caused by a moving vehicle, the BCG signal can be only identified and used when the vehicle is stationary and idling. Therefore, its necessary to employ advanced signal processing methods to filter the effective signal. The ECG signal, which is measured by insulated electrodes on the backrest of the car seat, remains stable for about 250 s and could be detected in a stationary position with varying speed. The baseline shift that was recorded during the first 250 s could be the result of a change in contact impedance due to a change in moisture in the garment. Another reason may lie in the varying distance between the body and the electrode surface, which decreases due to the time-dependent compression of the fabric layer. In non-contact biopotential scanning, signal quality depends on clothing thickness and pressure, and can affect the data in a negative way [25,43].

## 2. Overview of Already Existing Methods

Technology related to medicine has improved significantly over the past few generations, particularly in recent years. The development of various modern systems and methods became more advanced and less invasive at the same time. For example, the most advanced methods of vital sign monitoring include contactless respiration and heartbeat monitoring, which is a very interesting alternative to chest-strap monitors [44]. Another modern method is Doppler radar, which is also non-invasive [44,45].

This section presents the thorough overview of the most popular current vital sign monitoring methods, summarized in Table 2.

### 2.1. Car Seat Systems

**Figure 3:**
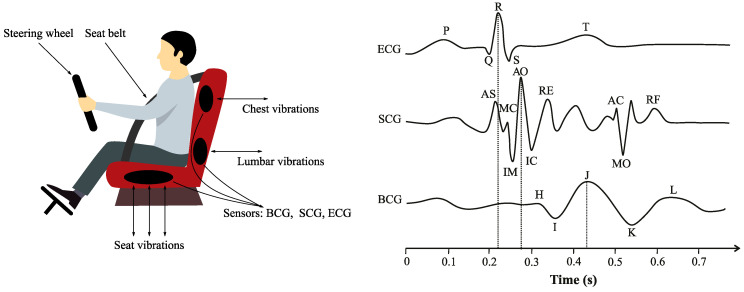
Location of sensors in the car seat and their signals.

Vital sign monitoring systems implemented into car seats are usually based on monitoring of cECG, BCG and SCG signals. The steering wheel (conductive part), backrest (capacitive part) and seat (capacitive part) are used as contact points of the electrodes in vehicles (see Figure 3). The grounding electrode is used to reduce common leads.

#### 2.1.1. cECG Electrodes

Since ECG measurement is nowadays used in clinical practice as a “gold standard” for measuring heart activity, many studies dealt with its implementation into vehicles. The ECG signal represents an electrical activity of heart cells during the heart cycle. It means that the ECG waveform is characterized by several significant points (see Figure 3): P wave representing the depolarization of the atria; the QRS complex as the result of depolarization of the ventricles; and the T wave, resulting from the repolarization of the ventricles. For ECG measurement purposes, different types of electrodes can be used, connected to the certain place of scanning. The capture points for monitoring ECG in cars together with the type of used electrodes are summarized in Table 3. As this section focuses on the sensors integrated into car seats, cECG monitoring methods are described further below. Table 3 summarizes the capture points for monitoring ECG from the designated sites.

The first implementations of cECG into others objects was presented by Park et al. [46]. They presented the successful integration of cECG electrodes into a chair. Authors compared the results of cECG with standard ECG measured by Ag-AgCl electrodes fixed directly to the subject body. Later Lim et al. [47] and Wu and Zhang [48] reported the integration of dry and cECG electrodes into beds. The experiments showed the dependence of the measurement accuracy on the position of the measured subject and the type of his clothing. Furthermore, the measurement appears to be significantly affected by motion artifacts. In 2006, the real application of cECG electrodes was introduced for the first time [49,50]; in their studies, researchers described the so-called Aachen Smart Chair. This chair is a modified office chair in which two solid copper plate electrodes are built. These electrodes are integrated into the backrest.

The integrated backrest electrodes were coated in a protective black acrylic paint for pure capacitive bonding. A similar design was proposed in [47].

Chamadiya et al. [26] introduced a concept of textile capacitive electrodes integrated into the conventional Mercedes seat. The electrodes were placed horizontally and laterally due to the limiting space. Unfortunately, lateral electrode placement was inadequate and resulted in reduced contact with the body and consequently also lower coverage.

Eilebrecht et al. [51] integrated a structure in which the multi-electrode design is located in the rear of the seat. The presented specific arrangement had a significant advantage—from among the different electrode pairs, the strongest signal could be freely selected. The system was, therefore, able to adapt to different heart axes as well as to the variable torso proportions.

In 2012, Schneider et al. [49] developed a custom framework capable of vital signs measuring inside vehicles. The system consists of two textile electrodes positioned horizontally in the lumbar region of the back and an electrode powered by a seat right above the two active sensing electrodes. The design was thoroughly tested and implemented in the Audi testing vehicle.

Jung et al. [52] introduced another monitoring system, which also deals with cECG. This system has two horizontal electrodes that are integrated into the car. The researchers placed the active measuring electrodes horizontally in the back of the seat. The electrode of the right leg was made of conductive fabrics and placed on the seat. Authors examined the complete HRV analysis without any heart disease.

A custom three-electrode system fully integrated into the car seat was announced in media in 2014. Leicht et al. [50] states that the localized water vapor can dramatically influence the measured SNR (Signal-Noise-Ratio) levels of cECG electrodes. They state that increasing local air humidity can lead to improved ohmic coupling (i.e., the coupling will no longer be exclusively capacitive), while it also allows the static charge difference induced with the tribo-electric effects to flow to the ground. This reduce the impact of tribo-electric power on capacity monitoring. They also proposed conductive textile electrodes which are vapor permeable and have placed silica gel chambers as a source or compensation of moisture behind the textile electrodes.

The team from the RWTH Aachen introduced a multi-electrode design integrated into Ford S-Max commercial test vehicle [51]. While solid metal electrodes remained in this design, their shape was changed to round, and the contract surface was reduced compared to the previous explored designs. In that study, patients were asked to exercise on a simulator with a capacitive seat ECG while also maintaining classic ECG monitoring for reference purposes [25].

Weder et al. [53] provided a textile breast belt which included a water reservoir used to periodically soak the electrodes. These solutions were fully integrated, but they did not provide any additional information concerning their effect on local moisture. Therefore, Leicht et al. [54] published a closed-loop concept for controlling local humidity feedback. Since the storage and release of water vapor on silica gel depend on the local water vapor pressure and temperature, a closed-loop temperature control was introduced as a method for releasing water vapor from the gel, leading to increased SNR efficiency.

A new system called the WARDEN^TM^ system [13] was introduced in 2016. It is a seat cover that includes regulated ground electrodes. These electrodes are built into the main electronic unit, which is located opposite the backrest.

In 2017, the Belgian research organization IMEC (Interuniversity Microelectronics Centre) introduced a novel design composed of six electrodes with circular capacitive electrodes in the backrest [55]. The overall design had a similar appearance to the one presented in [50]. The main difference lies in the additional integration of the radar sensors, which lead to sensor fusion.

#### 2.1.2. BCG and SCG Sensors

The BCG signal reflects the blood moving with each pulse around the vascular tree causing changes in the center of the body. Body micromovements are then generated by the back forces to maintain overall dynamics. The BCG is a record of mentioned movements. It is measured as a signal of displacement, speed or acceleration (in all three axes). The BCG longitudinal signal is a measurement of head-to-foot variations in the body. The BCG transverse signal represents front-rear (or dorso-ventral) vibration. The original BCG systems based on beds and tables focused on longitudinal BCG measurements [56,57].

The BCG makes it easier to monitor cardiac (as well as respiratory) activity. Usually focusing on external pressure or strain gauges, the BCG records vibrations, caused by the mechanical activity of the heart and lungs. The obtained diagnostic information is, therefore, significantly different from the conventional electrocardiogram used to record the direct electrical activity of the heart muscle [57].

The SCG signal reflects the mechanical vibrations of thorax caused by heart contraction and blood ejection from the ventricles into the vascular tree. The SCG signal is usually measured by a three-axis accelerometer when each of its components shows a specific pattern. In contemporary research, most studies focus on dorso-ventral components. However, one cannot omit the occurrence of biological information in longitudinal and lateral ones, including the acceleration vector trajectory [58,59,60,61,62,63,64].

Each heart contraction is depicted in BCG and SCG waveforms (see Figure 3). Each waveform is characterized by several peaks and valleys reflecting specific events of the heart activity. In BCG signal, three phases can be distinguished: pre-ejection (FGH complex), ejection (IJK complex) and diastolic (LMN complex) part of the heart cycle. J-wave, as a reflection of rapid ejection of both ventricles and blood acceleration in the descending and abdominal aorta, is the most prominent peak of the signal. SCG signal is characterized by these events: AS—atrial systole; MC—mitral valve closure; IM—isovolumetric contraction; AO—aortic valve opening; RE—rapid ejection; AC—aortic valve closure; MO—mitral valve opening; and RF—rapid filling.

Bruser et al. [23] explained in their work the application of nine different non-contact sensing modalities, which measured cardiorespiratory activity by sensing mechanical, bioelectric and thermal effects caused by various body disorders—specifically cECG, SCG, BCG, pulse oximetry, thermography, laser, radar methods, video motion analysis, as well as methods using high-frequency electromagnetic fields. When pressure or acceleration sensors are attached to the chest for the purpose of heart movements’ recording, it results, in particular, in BCG signal measurement [58,59,60]. For medical or diagnostics purposes, the sensors may be integrated into a bed, and they could be positioned either above or between mattresses (e.g., quasi-piezoelectric ferroelectric films), inside the mattress (optical sensors), on a slatted frame (using e.g., strain gauge sensors) or on beds (e.g., using a pressure sensor) [61,62].

In the 1950s, Scarborough et al. [58] presented a list of BCG measurement and defined the three measurement axes for the BCG translation records as longitudinal (head-to-foot, also called cranio-caudal), transversal (side-to-side) and dorsoventral (back to chest). It is noted that craniocaudal momentum is usually significantly greater than the transversal and dorsoventral component. A direct contact (mechanical connection) is required to reliably measure the BCG signal [1].

The early BCG recording systems were usually designed as longitudinal [63], which for a standing person corresponds with the z-axis [64]. Weights were also measured on this axis [65]. The early BCG recording systems were usually designed as longitudinal [63]. In this case, a standing person corresponds with the z-axis [64] on which weights are also measured [65]. A different approach is set by using bed-based BCG systems, when the signal is measured in combination of transversal and dorsoventral axes. Then, the z-axis is given by the dorsoventral one [66,67,68].

The BCG is nowadays becoming interesting again, mainly due to the development of simple and inexpensive instrumentation and the adaptivity of the whole technique, since it can be easily used outside conventional medical scenarios (e.g., sleep analysis) [37,69]. However, BCG signal shows much higher variability than the standard ECG signal, which is related to the body position of the patient. Thus, the BCG heart rete detection can be more challenging [69]. Despite these issues, Brüser et al. [70] developed a unique for detection of individual heart rates.

The driver monitoring based on BCG sensing has a large amount in common with the BCG monitoring implemented into chair [71]. In this position, the sensor is usually located on chest. Thus, the cranio-caudal BCG component is generally measured because of the possibility of insufficient connection between the torso and backrest, which can make the measurement of dorsoventral component problematic.

Early attempts to integrate the BCG sensors into car seats began about 11 years ago, when a successful placement of the BCG sensors in the passenger seat of the SMART car (Daimler A.G.) took place [71]. In this system, a qui-piezoelectric ferroelectret EMFiTM was used as a BCG sensor [72]. However, the presented data show that engine vibration might still prevent the reliable BCG monitoring in a running vehicle [11].

In order to fight with the driver’s fatigue (which allegedly causes 20%–35% of serious accidents, see in Figure 1 and Figure 2), the HARKEN project was introduced. It deals with monitoring of the driver’s cardiorespiratory activity, but without using the sensors directly attached to the patient’s body. The measuring method is based on smart materials embedded in the car seat and safety belt [12,73]. However, no other results of this BCG policy have been published so far.

In 2018, Wusk and Gabler [74] introduced an estimate of the cardiorespiratory function obtained using the BCG sensor in the passenger seat of the Ford Mustang, where the authors suggested using a “fluid-filled bladder attached to a solid-phase pressure transducer” as a sensor. The study contained the data collected from eleven volunteers, and the whole study was carried out in controlled laboratory conditions. As a result, the authors were able to extract both vital signs with fair accuracy.

Monitoring of vital functions in a car faces many problems with signal distortion due to interference caused by the vehicle and driver himself—for example, movement artifacts [75]. These artifacts are non-stationary and more prevalent in discrete sensory techniques [76]. Thus, the current research deals with their elimination using sensor fusion, which means combining multiple sensors at different locations, even different types of sensors [77]. This approach would be able:

- To compensate artifacts—by using advanced adaptive filtering techniques with a single sensor used to monitor the noise itself;
- To separate unique sources—by using algorithms based on statistical dependencies between each individual signal (such as independent component analysis);
- To increase the coverage rate—by using a set of sensors to measure the same vital sign, thus increasing the likelihood that this vital sign can be obtained reliably.

The improved coverage and robustness of vital signs estimation by fusing unobtrusive measurements was proved in 2015 [78]. In the experimental setup, the authors actually combined camera-based information (a webcam for BCG and pulse wave) and information about a one-dimensional signal (BCG in the seat).

Modern research in BCG and SCG measuring techniques also deals with wearable monitoring systems, allowing continuous vital sign recording during everyday life [56], in any environment or under stress [1]. In this case, a three-axis accelerometer is generally used, which can be mechanically attached to the body either by adhesive, plastic fastening or textile [79]. The SCG assessment using an external three-axis MEMS accelerometer mounted on the left clavicle attached to an intelligent garment with textile ECG electrodes to obtain simultaneous three-axis SCG and single-lead ECG records was thoroughly tested [80]. This concept was subsequently improved by Di Rienzo et al. [81], who designed an integrated vest equipped with a MagIC-SCG accelerometry device. Omer T. Inan [56] summarized in his study the key achievements and limitations of modern BCG systems, which are presented in Table 4.

#### 2.1.3. Optical Sensors

Heartbeat was examined for a long time mainly based on the sound of the heart. This basic diagnosis, often carried out with a stethoscope, is nowadays expanded, and other additional components, such as force, frequency and sound pressure, are often extracted as well. Optic sensors are often used as an alternative approach. A device based on Fiber Bragg Grating (FBG) monitor heart rhythm by measuring the acoustic pressure of the muscle itself. D. Gurkan et al. even introduced a modified version that leverages BCG as well—the captured sound signal is converted into an electrical by a reflex-sensitive FBG sensor [87]. This design was verified in a specific scenario. The FBG sensor was glued onto a subwoofer. During a recording of various heartbeat sounds, specific spectral changes were recorded at sampling frequency of 250 Hz. The system showed an increased sensitivity to various heartbeat strength, frequency and time content of the oscillation. The designed non-invasive FBG sensor proved to be a reliable alternative approach for the extraction of various information from the heart sounds, and even managed to measure heart strength. Non-invasive methods are currently explored by various researchers, as they have a number of benefits—mainly for patients [88,89].

A description of the design and manufacture of smart fabric based on the FBG field was at first described by D.L. Presti et al. [90]. Their study showed the assessment of the feasibility for monitoring respiratory parameters (i.e., respiratory rate, respiratory period and inspiratory and expiratory period) and heart rate in healthy volunteers in two positions (standing and lying down). The increased number of FBGs was incorporated into this system with respect to previously developed prototypes. The main goal was to improve its accuracy in estimation of these parameters. The testing was carried out in order to determine whether the proposed solution made it possible to improve the measurement of respiratory volume exchanges in new scenarios, such as sports medicine, or activities such as walking, running and cycling.

The Complete Ensemble Empirical Mode Decomposition with Adaptive Noise (CEEMDAN) and sensor fusion for automatic heart rate detection from a mat with built-in FBG sensor arrays was investigated by Sadek et al. [91,92]. The fusion process was performed in the time domain by averaging the sensor readings for each sensor field. Afterwards, the CEEMDAN was applied in order to gather internal beat intervals. The experiments were performed on 10 human volunteers lying in two specified positions on the bed for 20 min. The overall system performance was evaluated based on the reference ECG signals. The fusion of the sensor and CEEMDAN proved to be resistant against movement artifacts caused by random body movements [91].

### 2.2. Steering Wheel Systems

The implementation of sensors integrated in a steering wheel allows non-invasive heartbeat and pulse wave measurement. Modern systems, where a steering wheel is applied, do not require neither particular preparation nor conductive gel or paste [30,93].

One of the first research goals was to place the electrodes on the steering wheel in order to quantify driver stress levels, when investigating the load on the driver and the load response based on HRV obtained with a pair of dry steering-wheel ECG electrodes [94]. Similarly, in the same year, Lee et al. [95] reported their studies similarly using steering wheel with integrated dry electrodes made of copper tape. For the study purposes, three students were admitted as test drivers and asked for directions for 16 km for 40 to 50 min.

Shin et al. [96] and later Jung et al. [52] introduced electrodes placed on the steering wheel made of conductive fabric. Choi et al. [97] introduced a steering wheel, which is covered with a conductive dry material of the fabric-based electrode, was manufactured by galvanic plating. 

In parallel, a multi-sensor system consisting of infrared thermometer and reflective optical sensor for measuring pulse oximetry and skin temperature implemented in the S-Class Mercedes was published by Heuer et al. [98]. In parallel, D’Angelo et al. [8,9] demonstrated a multi-sensor system integrated into the steering wheel of the BMW 730d test vehicle, which enabled pulse oximetry and conductive ECG scanning. They applied a similar design to the one proposed by Jeong et al. [94], which integrated the electrodes into the steering wheel, the gear shift and the left armrest. However, the exact location of the electrodes was clearly stated [98].

Gomez-Clapers and Casanella [99] presented a steering wheel equipped with the ECG demonstration meter based on dry ECG electrodes and wireless communication. Similarly, Silva et al. [100] demonstrated that steering wheel equipped with dry Ag/AgCl electrodes managed to obtain the ECG measurements of a similar quality to traditional electrodes.

Another study [100] presented a custom design of a steering wheel, which contained a set of two conductive fabric electrodes, similar to those used by Jung et al. [52], where the electronic circuit design was based on the MSP 430 microcontroller and the Chipcon CC2420 RF transceiver. Apart from measurements, the system offered a way to wirelessly transfer data in a 2.4 GHz band.

ECG monitoring based on a steering-wheel with integrated electrodes can be a good candidate for heart rate monitoring but requires that both the driver’s hands interface with conductive parts of the steering wheel. Significant steering movements along with grip position changes, and the relatively common one-handed steering habit causes a significant problem for this technology and negatively affects the data quality [101].

A different design way was proposed by Matsuda and Makikawa [102] who suggested using a conductive steering wheel in combination with a capacitive electrode placed in the driver’s seat. This system managed to overcome the common one-handed steering custom and its consecutive problems. This proposed system also combined conductive measurement concepts with capacitive sensing, which was later called hybrid sensing. This hybrid method was also suggested by Baek et al. [103] as a useful addition to redundant scanning and sensor fusions. The idea of steering wheel electrodes measuring against a capacitive electrode in the seating area was once again introduced and researched by Xu and Ta [104].

### 2.3. Helmet Systems

Analysis of EEG signals is a very complex task which is even more difficult when the main system has to communicate with other various subsystems to source data or maintain proper [76,105,106,107]. That is due to the non-stationary character of these signals, their susceptibility to various artifacts (external and internal) and other disturbances [76,106,107]. The EEG signal is usually analyzed and evaluated in various frequency spectra: Delta rhythm (0.5 ≤ f < 4 Hz) is a symptom of deeper stages of sleep; Theta rhythm (4 Hz ≤ f < 8) is typically present in the initial stages of sleep; Alpha rhythm (8 Hz ≤ f ≤ 13) is the main manifestation of the resting brain activity, while Beta rhythm (13 < f < 30 Hz) is present in nervous or anxious subjects.

As mentioned in the previous part of this work, the number of annual traffic deaths is constantly growing and is the leading cause of death of people in the 5–29 years bracket. As driving is a very complex task (from the biomedical point of view), it requires a large amount of concentration. Any distractions or health problems can affect driving in a negative way resulting in an accident. That is why advanced driver assistance systems—e.g., based on EEG—were introduced [105].

Application of the EEG data as a vital sign tracking while driving can be a very useful tool. Sudden changes in sensory, motor and cognitive functions are strongly related to the driver’s age, and there is even some evidence stating that lower Alpha and Beta frequency ranges correlate with slower reaction time while driving [108]. Cognitive state of drivers can be analyzed with the implementation of inter alia channel-wise convolutional neural networks (CCNN) [109].

Various inexpensive devices were tested to measure and analyze EEG recordings [110,111,112,113]. One of the first commercial, inexpensive devices applied for EEG was a headset from NeuroSky Mind-Wave, which was supposed to capture different levels of EEG [114,115]. In addition, a limit switch electrical circuit, which controls car engine start, was designed and connected between the seat belt and the ignition system. In this early solution, the Arduino microcontroller was used along with its software as a signal processing unit to control various safety systems of the vehicle. The experimental results have shown that the system was able to reliably increase the safety of drivers [116,117].

Consumer-grade level EEG devices are becoming more and more popular. Among such systems are the above mentioned Neurosky headsets (Mind-Wave and MindWave Mobile), EPOC and FLEX from Emotiv, Ultracortex (Open BCI), ENOBIO (Neuroelectrics), DSI (QUASAR), interaXon, Smarting (mBrainTrain) or Quick (Cognionics) [110,112,113,118]. Especially Emotiv EPOC is a very popular and inexpensive solution which, despite the appearance of newer versions (Flex and Insight), is still being applied in numerous BCI-related projects [107,110,112,119]. MindWave Mobile and Yband also stand over other numerous inexpensive headsets, mainly due to their price and reliability. However, their implementation is very limited as they are capable of measuring from only one or two electrodes placed on the frontal lobe [111].

Besides age and cognition level of the driver, another major reason causing car accidents is driver drowsiness, which became a major focus of safety research in 2005, when EEG was applied in order to detect the immediate state of the driver [120].

### 2.4. Camera Systems

While the seat-integrated techniques (ECG, cECG and BCG) have been used extensively in the automotive environment [121]; camera-based techniques, although not studied at a high level of readiness to the same extent as seat-integrated techniques, also remained popular [122,123]. The main problem probably lies in their relatively high costs and a potential conflict with drivers—the camera can be taken as a violation of their privacy [123,124].

**Figure 4:**
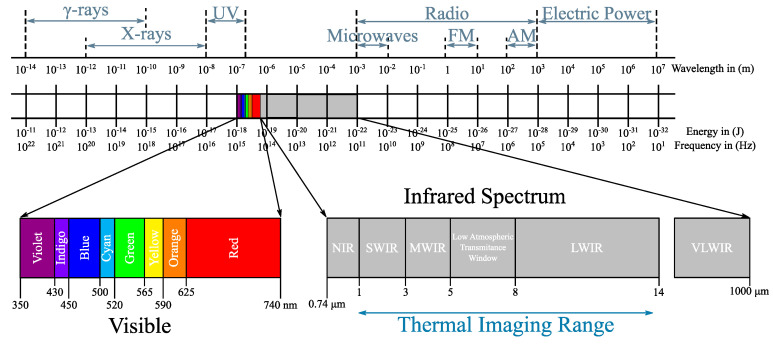
Frequency ranges—visible, near-infrared, far-infrared—usable for optical monitoring techniques.

The advantage of the camera-based vital sign measuring system is the imperceptible contactless transmission of information from the driver or passenger. Optical modalities potentially offer unobtrusive remote sensing, which is attractive for automotive applications, especially in modern automobiles, where cameras are used to detect external obstacles and so on. In the recent years, there was a boom in camera technologies and their application in vital sign monitoring. Figure 4 shows frequency spectra suitable for cameras [1].

**Figure 5:**
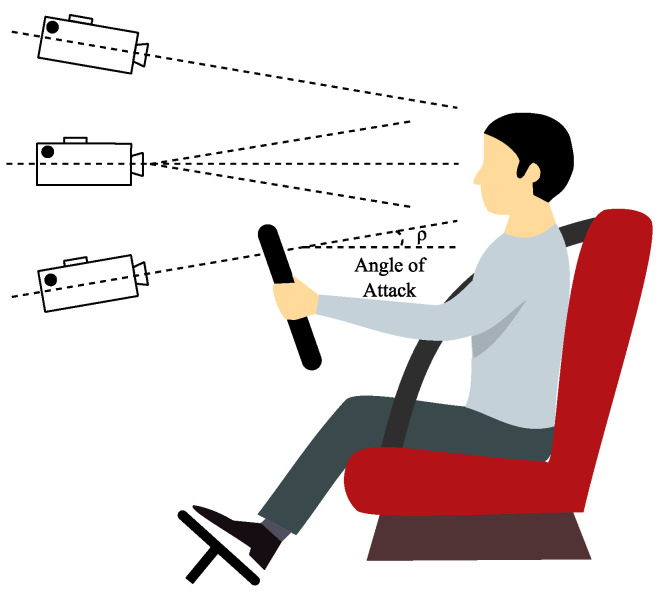
The locations for the cameras. Locations can be distinguished by the angle of attack ρ.

Three frequency bands are suitable for monitoring the driver’s vitals: visible light (350–740 nm), near infrared light (750–1000 nm) and far infrared light (1 μm–1 mm). Within the visible and near infrared range, light can freely interact with living tissues. The light can move through the tissue in a direct path (as in transmissive pulse oximetry), or it can also scatter. In fact, in the case of the skin tissue, the scattering tends to be the dominant effect of the tissue-to-photon interaction. As with any optical method, the driver or passenger tracking via an integrated camera requires constant supervision. As the current cockpit design requires a clear view for the driver, the following possible camera locations have been explored (see Figure 5) [1].

### 2.5. Radar Systems

Radar devices can be used in non-intrusive situations to monitor vital signs. Accurate simultaneous estimates of heart rate and respiratory rate are possible by detecting and extracting body movement associated with physiological activity. Most research focuses on anterior monitoring of superficial chest motion. Schires et al. [125] demonstrated the feasibility of back-monitoring vital signs using pulse radar. They demonstrated the physiological movements in the body by demonstrating the attachment of the radar to a car seat.

For proper monitoring of post-contact applications, it is crucial to eliminate accidental body movement. When using Doppler radars, the suppression of random body movement is usually performed by phase measurements, which are obtained from two opposite sides of the human body. In their article, Munoz-Ferreras et al. [126] proposed the method which leverages two frequency-modulated continuous waves of radars to solve the phenomenon of random body movement.

Lohman et al. [127] proposed a new signal processing method for continuous heart rate monitoring using ultra-wideband pulsed radar. The processor can even reliably calculate both subject speeds at distances of up to 2 m. Speed determination was based on autocorrelation in addition to several enhancement techniques.

Leem et al. [128] proposed a new algorithm, which estimates vital signs even when random movement occurs. The entire fast detection area of vital signs was analyzed and signals, obtained while driving, provided useful information regarding drivers’ condition. These signals were then divided into sub-signals, and then, the desired vital signal was generated with the implementation of the correlation method. In this way, the driver’s vital signals could be monitored non-invasively, which could be potentially used for detection of driver drowsiness. The authors also discussed robust monitoring of vital signs using pulsed radio ultra-wideband (IR-UWB) radar.

Zito et al. [129] developed the custom sensor based on 90 nm CMOS technology. They experimentally characterized a UWB radar sensor for the respiration rate monitoring. The radar test chip was used for non-contact detection of respiratory activity in adults and children. Field tests have shown that the UWB radar sensor detected the person’s respiratory rate associated with the chest movements up to units of centimeters, allowing continuous non-invasive follow-up monitoring of the hospital patients and other persons at risk of apnoea obstruction.

### 2.6. Other Sensors

Besides the sensor types described above, other possibilities are often discussed in the literature.

Among them is a preventive system mounted on the steering wheel which uses a magnetic sensor built into the seats of cars and trucks developed by the Tsuchida et al. [130]. The measuring system is based on the implementation of build-in torsion bars in the driver’s seat. The authors tried to monitor drivers by measuring the differences in the magnetic properties of these torsion bars—the torsion voltage of the magnetic sensor depends on the stress caused by the sitting driver. The measuring unit registers changes between voltage induced by the excitation coil and registered by pickup coils.

Real-time eye movement classification offers a very effective way of human–machine interaction [131,132]. However, these systems require the sensors to be placed around the eyes, which might be distracting and cause significant discomfort [131]. In one among numerous studies, Zhao et al. [133] used two EEG sensors located above the temporal areas to classify real-time eye blinks and five distinct classes of eye movement direction. The gathered data were then processed, and a set of discernible time series functions was extracted. The presented solution reached up to 85.2% in case of accuracy and 77.6% in case of sensitivity. The used algorithm with its high accuracy and low latency clearly shows that it is an effective solution for reliable detection and classification of eye movement. Desk-mounted eye tracking sensors which use human–machine Interfaces and Interaction to extract features already exist [131].

Electrical sensors are also widely used. They allow the monitoring of respiration and heartbeat rate and can be also used for non-invasive, contactless radar sensors. The early concepts of these systems are very promising, but their real-world implementation is significantly more difficult as the simultaneous respiration and heartbeat detection is currently impossible [134].

## 3. Interference and Signal Pre-Processing

Vital sign monitoring plays a crucial role in both prevention and diagnosis of various health problems [135,136]. As more and more people pay attention to their health condition, various measurement systems are developed, in particular, following the so-called “wearable” concept. The problem with these devices is that they can be somewhat uncomfortable for patients as they require direct physical contact with the body. Contactless solutions are, on the other hand, prone to various interferences and artifacts, and therefore, are less efficient [134].

### 3.1. Noise and Interference

**Figure 6:**
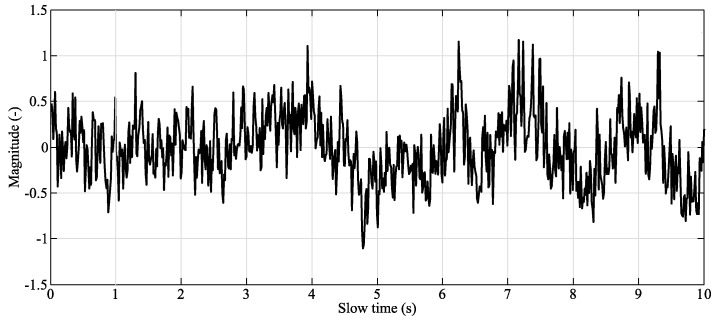
Noisy vital signal obtained by conventional algorithm—during motion period.

In the literature, there are many well-known algorithms for the measuring of human vital signs [137,138,139]. The vital signs, as with other biomedical data, are prone to the occurrence of various internal and external artifacts, which could be related to some specific interferences [76,107]. Moreover, measurement of vital signs while driving is a different challenge, since there are various body movements [140,141]. The occurrence of the interference in examined or recorded data affects their quality in a negative way (see Figure 6 [128]) and makes the whole analysis process much more difficult [49].

Among the most important interferences is the one associated with the powerline, which is 50 Hz for Europe and 60 Hz for the US. It can be easily compensated by the basic Notch filter [88,142]. In the case of the cECG recordings, there is an occurrence of both inter-modal and intra-modal interferences, which do not affect the quality of the recording and does not interfere with the vital sign monitoring [88].

Other interferences are often connected to the driver’s movements or antenna designs (for wireless data transfer) [143]. In addition, other signals, such as those caused by the background (generated by other passengers or the vehicle—e.g., engine noise), can lead to major unnecessary interferences [144]. Table 5 summarizes an occurrence of different types of interference in the measured signals using methods discussed in this paper.

The researchers in [139] discussed the problem of vital functions of a non-stationary character, but their approach was very simple—they were able to detect body movement and block the measurement of vital signs until body movement stopped. Such approach might be advantageous for certain situations but might lead to other significantly dangerous scenarios. In [128], the team managed to not only detect body movement, but also to measure vital signs during the period of movement.

Motion artifacts can also significantly impair the signal quality due to the varying connection between sensors and the subject. One way to address this challenge is to combine multi-channel evaluation using sensor fusions. For a robust and accurate sensor interconnection, it is essential to analyze the effects of motion in various ways. Antik et al. [78,128] introduced a custom experimental setup, combining cECG, reflex pulse oximetry, impedance sensors and BCG sensors. A series of early experiments was carried out to quantify movement artifacts. In addition, a set of predetermined exercises was carried out by testing subjects to perform a cardiorespiratory monitoring. A shape-based SNR was used to quantify and analyze the effect of human body movement on performance of different sensors. The work also shows the optimal combination of sensors and a concept of future fusion methodology [145].

### 3.2. Advanced Signal Processing Methods

**Figure 7:**
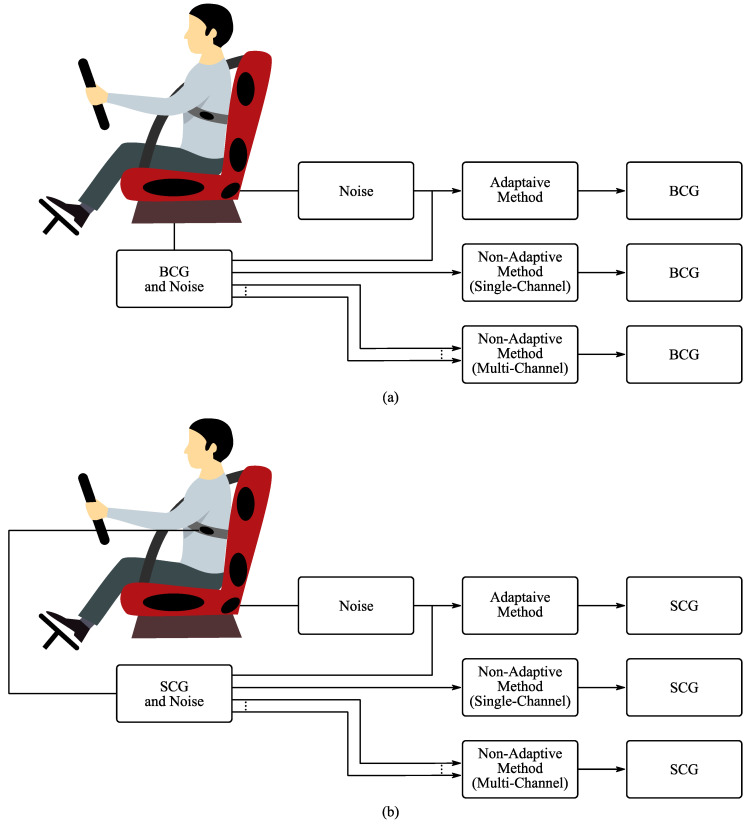
Overview of signal processing methods and car seat. (**a**) BCG signal processing and (**b**) SCG signal processing.

The signals measured using the BCG and SCG sensors are influenced by different types of interference mentioned before—mainly by vibrations and movements of driver. To extract the desired information of the driver’s physiological functions, it is necessary to use the advanced methods of signal processing. Generally, these methods can be divided into adaptive and non-adaptive. Although non-adaptive methods require only specific sensors for measuring the BCG signal with noise (or SCG signal with noise), adaptive methods need at least another one sensor set for measuring the interferences (reference signal), as shown in Figure 7.

The most commonly used adaptive methods include techniques based on neuro-fuzzy inference systems (ANFIS), Least Mean Squares (LMS) and Recursive Least Squares (RLS) algorithms. Non-adaptive methods can be divided into single-channel and multi-channel methods, based on the number of input signals required. The well-known single-channel methods are, for example, filtration based on Fast Fourier Transform (FFT), Discrete Wavelet Transform (DWT) and Empirical Mode Decomposition (EMD). Multi-channel methods comprise the set of methods called Blind Source Separation (BSS), including Independent Component Analysis (ICA) or Principle Component Analysis (PCA) [107,144].

Hybrid methods present another possibility to properly eliminate the artifacts and interferences, as they combine the aforementioned methods. Many researchers often use the ICA method as a pre-processing for some adaptive algorithm, and the single-channel non-adaptive method for smoothing of the desired signal. The overview of the discussed signal processing methods is shown in Table 6.

The most frequently used adaptive methods are based on LMS-based noise cancellers. Although they tend to suffer from a higher delay in comparison to other adaptive methods. However, this delay can be reduced using pipelined filtering [146]. Manjula et al. [147] achieved up to 80% of noise reduction (mainly vibrations) from the simulated BCG signal. The adaptive noise cancellation using the LMS algorithm for elimination of floor vibrations was proposed in [148]. The authors monitored BCG signal measured by bathroom scale and filtered the vibrations using a reference seismic sensor (geophone) placed on the floor next to the scale. This approach can increase BCG measurements robustness in home monitoring applications as well as in ambulances or other vehicles. Using the similar methodology, the electromyogram signal taken from the feet of the subject can be used as a noise reference for motion artifacts reduction using adaptive filtering [149]. Yang et al. [150] extracted SCG data using LMS adaptive algorithm from moving subjects with a significant improvement of signal quality.

Non-adaptive methods have a great potential for BCG signal processing. Etemadi et al. [151] used EMD for denoising of SCG signal obtained using wearable measuring system, which has been successfully used in the past to remove baseline wander and other noise from the ECG waveform. The SCG signal components that were related to movement were shifted from the components related to heartbeats, so the SCG waveform morphology was improved. Postolache et al. in 2010 [152] introduced a multi-sensing system with BCG sensors embedded on a smart wheelchair. They used a combination of ICA and WT for signal processing and estimation of heart rate and respiratory rate. They achieved a good accuracy during filtration by mentioned methods. Manjula and Sharma in 2017 [153] also used the ICA method for BCG denoising. This method was used for preprocessing and for dividing source signals. In next step, k-means was used for the determination of which components corresponded to noise, and suppressed them. The proposed method provided a good accuracy and outperformed other conventional BCG processing methods. Javed et al. in 2014 [154] and 2017 [155] used combination of PCA and EMD methods for BCG processing. This method was named PCA-EMD. These studies showed that noise in BCG signals could be effectively eliminated by the proposed approach. However, higher computational cost is a main limitation of this approach. The main advantage of mentioned methods is that they do not require any reference signal and no prior information, or any assumption, is needed to perform quality filtration.

Table 6 represents the comparison of various signal processing methods, covering individual important parameters and their optimal settings. As these methods offer distinctive advantages and also disadvantages, their fusion could offer other significant gains. Hybrid systems combine multiple algorithms to gain significant strengths in different fields of signal processing. These systems are currently explored and will definitely surface more often in the future.

Computational costs is another significant factor for future implementation of BCG and SCG signal processing methods. Modern vehicles operate with limited CPU/GPU performance and RAM space. Any computationally intensive operations could influence the successful operation of other, often much more important, systems. Therefore, the system has to take the limited resources of vehicles into account and work around the deployed hardware. Modern carmakers are already replacing typical electronic control units (ECUs) with centralized computing platforms, which tend to offer a much higher performance. However, these systems must be robust to operate in large temperatures and altitude ranges. A subsystem, specially designed for processing and evaluation of BCG and SCG signals could offer a better way to continuously gather drivers’ vital signs, while not stressing the vehicular central unit. Table 7 and Figure 7 represents overview of individual parameters and most often used optimal values for various signal processing methods.

## 4. Examples of Patents

One of the most interesting patents was the one made by Elrod et al. [156], who patented a function monitoring system with sensors mounted into the child seat, which manage to monitor one or more occupants’ health conditions. The applied processor generates the status signals, and the implemented transmitter wirelessly transfers the status signals to a remote receiver. The signaling device connected to the receiver produces at least one sensory (e.g., visual, audible, tactile) output based on status of the received signals.

Another patent where a sensor was integrated into the child seat was presented by Bellamy [157]. A sensor unit was mounted on the housing. The sensor managed to detect vital signs in addition to another parameter—weight. The vital signs include body temperature and heart rate. The sensor also detects ambient air temperature adjacent to the child seat. The display is mounted on the child safety seat and is electrically connected to the detection unit, showing all measured parameters. The power supply is mounted in a housing and is electrically connected to the sensing unit and the display.

Another invention, which can be used to conduct analysis of the driver (land, water and air vehicles) before commencing driving, provides information regarding stress level and health status throughout the driving, even working over extended periods of time. Such system was developed and patented by Tausch et al. [158]. The device analyzes and evaluates HRV in the time domain and frequency domain using Fourier transform. It is based on a signaling system similar to the one applied in traffic lights. The red colors indicate the expected state of stress and health, while the greens mean best health.

## 5. Discussion

With the ongoing era of Internet of Things (IoT), various smart technologies are combined or connected to each other, enabling their integration and interaction. These data are nowadays very easily shared and exchanged. New methods for road-safety improvements have been introduced with the implementation of IoT applications and they often enable the monitoring of the driver’s state, counting of the number of passengers or even detecting an unattended child’s presence in a car on a hot day [159].

The future of vital signs measurement in car seats lays in contactless sensors. Based on modern signal and image processing methods, many of the technologies described in this paper will be implemented in smart cars [160]. It might work in a way that when critical health condition is detected—e.g., asthma or heart attack—the car will stop itself and call the emergency service. Furthermore, when the system recognizes that the driver has consumed alcohol, drugs or other addictive substances, the system will not allow him to drive. As the modern cars will be equipped with location information and, of course, a communication interface, it will be easier for medical services to track the driver of individual vehicles. Modern systems will also be able to sense emotions, so the system will alert the driver that he is not in a suitable condition to drive. Vehicles will also have sophisticated anti-microsleeve systems, while also having sophisticated fatigue detection systems. In the future, sensors in the car might also become a part of a larger telemedicine system—remotely monitoring patient health [136].

However, the moving vehicles generate a large amount of interference which influence multiple systems such as those based on BCG, ECG, etc. The signal can be negatively affected by noise and interference coming from several sources including sensor itself. Motion artifacts, generated by either body motion or vibrations coming from the road [11], are still a major limiting factor and can be definitely reduced by the implementation of various sophisticated filtering methods. Another issue, which has to be faced by future manufacturers is the ethical aspects of selected monitoring methods [161].

## 6. Conclusions

This work mainly focuses on a thorough review of inconspicuous and contactless sensors used to monitor vital functions. These systems will definitely be introduced into practice in the future (in the car seat, seat belt, steering wheel, etc.), but their advanced implementation is a subject of further research. Many technologies have been proposed based on various physical principles. The results presented in this work are mainly focused on their potential car implementation, but they can be easily transferred to other areas, including military applications, aircraft or even to personal healthcare (telemedicine) applications [11,161].

Techniques for the integration of ECG, cECG, BCG and SCG into seats have been applied extensively in the automotive environment; meanwhile, camera-based techniques, although already at a high level of readiness, have not yet been explored to the same extent. This may be due to their relatively high cost and possible conflict with the driver’s privacy.

Contactless sensors available today are still limited by motion sartifacts. Some specific technologies have even bigger challenges (such as tribo-electrics for cECG and changing light conditions in the visible and NIR frequency ranges). Sensor–signal fusion can be a way to overcome many of these limiting factors, as it can be also used to motion artifact elimination. These technologies require multiple sensors and simultaneous multi-channel measurements with redundant sensor deployments.

## Author Contributions

Conceptualization, M.S., A.K.-S., M.L., R.J. and L.D., Methodology, M.S., A.K.-S. and M.L., Software, M.S., Formal Analysis, R.J. and L.D., Investigation, M.S., A.K.-S. and M.L., Resources, R.M. and P.S., Data Curation, M.S., Writing—Original Draft Preparation, M.S., A.K.-S. and M.L., Writing—Review & Editing, M.S., R.J., L.D. and M.L., Visualization, R.M., A.K.-S. and P.S., Project Administration, R.M. and P.S., Funding Acquisition, R.M. and P.S.

## Figures and Tables

**Table 1:** Sensor’s distribution.

Non-Attached Sensor	Attached Sensor
eye movement monitoring sensors	helmet
head movement monitoring sensors	electrocardiographic (ECG) electrodes
mouth movement monitoring sensors	galvanic skin response sensing monitor (Electrodermal Activity)
vigilance monitoring system based on the driver’s behavior	respiration belt
accelerometer capacitive electrodes	pulse sensors
infrared sensors	
ultrasonic sensors	
Harken sensor	
optical sensors	

**Table 2:** Overview of the most recent sensor-based methods.

Location/Type of Sensor	Principle
car seats	monitoring of vital signs, such as cECG, BCG, seismocardiography (SCG) or all at once;
steering wheel	cECG or pulse wave monitoring via conductive systems;
front camera	monitoring of the driver’s face and evaluation of signs of fatigue, such as frequent blinking, eyes closing or head dropping;
back camera	monitoring of horizontal and vertical traffic signs, detection of lane deviations and traffic restrictions; in cooperation with the front camera, tracing the driver’s view and determines whether he has registered the vertical traffic sign with the view; detection of time of collision with an obstacle in front of the vehicle;
helmet	included in a test phase; monitoring of the driver’s condition through the EEG.

**Table 3:** Summary of the sensing points and the electrodes type used.

Place of Scanning	Electrode Type
driving-wheel	dry and conductive fabric electrode
conductive driving-wheel	capacitive electrode
armchair and bed	capacitive electrode
office chair	grounding and reference electrode
car seat	capacitive electrode
breast belt	conductive electrode
car seats via bluetooth	circular capacitive electrode

**Table 4:** Classification of modern BCG systems.

Modern BCG System	Measuring Axis	Key Benefits/Successes	Challenges/Constraints
Accelerometer in center of weight [82]	3-axis	- Characterized 3D BCG vector - Measured 3D BCG in microgravity	- the need for weight reduction—either in space or with dry immersion
Bed and chair [70,83,84]	Longitudinal or out of the plane	- Minimal movement artifacts (usually) - Easy to integrate into home and everyday life	- Changes in sleep position may affect signal quality and morphology - It is difficult to pair BCG with other physiological measurements (e.g., ECG)
Monitor vital signs on the ears [85]	Primarily longitudinal	- ECG can be measured simultaneously - Miniature, potentially cheap system	- Head position may affect signal integrity - Repeatability to be assessed
Weight [35,36]	Longitudinal	- Correlation with CO/contractility changes - obtaining multiple physiological signals in addition to BCG	- Postural differences between measurements may affect signal integrity - Motion artifacts must be automatically detected and mitigated
Vest MagIC [37,86]	Primarily longitudinal	- Correlation with CO changes - Obtain multiple physiological signals in addition to BCG	- Signal variation based on sensor position - Motion artifacts must be automatically detected and mitigated

**Table 5:** Interference occurring in the measured vital signs data.

	ECG/cECG	BCG/SCG	Ultra-Wideband Radar	Fiber Bragg Grating
Powerline	High	Low	Low	Low
Motion	Medium	Medium	High	Medium
Background noise	Low	Low	Low	Low
Vibrations	Medium	High	Medium	High

**Table 6:** Overview of signal processing methods.

Method		Overall Performance	SNR Improvement	Computational Cost	Real-Time	Implementation Complexity
Adaptive Methods	ANFIS	High	Medium	High	No	Complex
	LMS	Medium	Medium	Medium	Yes	Simple
	RLS	Medium	Medium	Medium	No	Simple
Non-Adaptive Methods (Single-Channel)	FFT	Medium	Medium	Low	Yes	Medium
	DWT	Medium	Medium	Low	Yes	Medium
	EMD	High	Medium	High	No	Medium
Non-Adaptive Methods (Multi-Channel)	ICA	Medium	Medium	Medium	No	Medium
	PCA	Low	Medium	Low	Yes	Simple
Hybrid Methods		High	High	Medium/High	Yes/No	Medium/Complex

**Table 7:** Overview of individual parameters and most often used optimal values for various signal processing methods.

Method	Parameters	Description	Optimal Values
ANFIS	Epochs Mf Shape	Number of epochs Number of membership functions Type of the membership function	[10, 20] 6 Gaussian/bell-shaped
DWT	n Ψ	Decomposition level Maternal wavelet	[3, 7] Daubechies/Symlet
EMD	IMFs SD	Number of intrinsic mode functions Size of the standard deviation (stopping criterion)	15 [0.2, 0.3]
LMS	M/N µ	Filter length/Filer order (N=M−1) Step size (convergence constant)	[1, 100] [0.00001, 0.1]
RLS	M/N λ	Filter length/Filer order (N=M−1) Forgetting factor	[1, 100] [0.9, 1]
ICA, PCA	Input Output Iterations	Number of input channels Number of outputs components Norm of the iterative step	Minimally 3 Minimally 3 1000
